# Ruptured Amoebic Liver Abscess With Empyema, Venous Thromboembolism and Bronchopleural Fistula: A Case Report

**DOI:** 10.1002/ccr3.71969

**Published:** 2026-02-10

**Authors:** Nur H. Ahnal, Siti N. Mohd Nawi, Wan S. Wan Ghazali, Alwi Muhd Besari, Hafsah Sazali, Fathin Hadi

**Affiliations:** ^1^ Department of Internal Medicine Hospital Pakar Universiti Sains Malaysia Kota Bharu Kelantan Malaysia; ^2^ Department of Internal Medicine, School of Medical Sciences Universiti Sains Malaysia Kota Bharu Kelantan Malaysia

**Keywords:** amebiasis, amoebiasis, bronchopleural fistula, infectious disease, liver abscess, liver empyema, parasitic infection

## Abstract

Amoebiasis, a gastrointestinal infection caused by *Entamoeba histolytica* , is the third leading cause of mortality worldwide among parasitic infections with over 100,000 deaths annually. Apart from dysentery, it can manifest as extraintestinal disease, most commonly liver abscess, and rarely pulmonary, cardiac, and brain involvement. This case demonstrates the challenging management of a complicated amoebic liver abscess (ALA) with pulmonary involvement and venous thromboembolism. A 26‐year‐old male presenting with pleuritic right hypochondriac pain, fever, and dyspnoea underwent hepatobiliary ultrasonography (US) which showed a liver abscess. Computed tomography (CT) of the thorax and abdomen revealed a ruptured right liver abscess with infra‐diaphragmatic intrathoracic extension and right lung empyema with the presence of inferior vena cava (IVC) and right hepatic vein thrombosis, and right pulmonary embolism. He underwent placement of a percutaneous pigtail thoracostomy catheter for drainage of right lung empyema which was complicated with a bronchopleural fistula. Amoebiasis serology was later reported positive for immunoglobulin G (IgG). The patient was treated with medical therapy which included tissue and luminal amebicides and warfarin. Video‐assisted thoracoscopy surgery (VATS) was performed to visualize and repair the bronchopleural fistula. Follow‐up CT revealed significant reduction in size of the right lateral intrathoracic and liver abscess. This case underscores the importance of awareness of pulmonary involvement in ALA and the associated risk of venous thromboembolism as early detection facilitates effective management and reduces mortality.

## Key Clinical Message

- ~10% of individuals infected with *E. histolytica* develop invasive amoebiasis, with colon and liver being the most frequently affected organs.
- Medical therapy of invasive amoebiasis consists of tissue amebicides followed by luminal amebicides.
- Early recognition of complications including pleuropulmonary involvement and venous thromboembolism is crucial for prompt and effective management.

**Abbreviations**
AFB: acid‐fast bacilli
ALA: amoebic liver abscess
ALP: alkaline phosphatase
ALT: alanine transaminase
Anti‐HCV: anti‐hepatitis C virus
APTT: activated partial thromboplastin time
AST: aspartate transaminase
CT: computed tomography
HBsAg: hepatitis B surface antigen
HIV Ag/Ab: human immunodeficiency virus antigen/antibody
IgG: immunoglobulin G
INR: international normalized ratio
IVC: inferior vena cava
MTB: mycobacterium tuberculosis
PCD: percutaneous catheter drainage
PCR: polymerase chain reaction
PT: prothrombin time
US: ultrasonography
VATS: video‐assisted thoracoscopy surgery
WBC: white blood cell

## 1 Introduction

*Entamoeba histolytica* is the pathogenic protozoan in amoebiasis, a leading cause of diarrhea worldwide [1]. The life cycle of *Entamoeba histolytica* involves consumption of fecal‐contaminated food and water that reaches and invades small intestinal epithelium to enter the liver via the hepatic portal circulation [1, 2]. It affects men 10 times more commonly than women, mostly in individuals between the ages of 18 and 50 [3, 4]. The prevalence is high in tropical and subtropical regions of Central and South America, Asia, and Africa. In South‐East Asia, it is reported mainly in areas with poor hygienic conditions and lack of effective sewage systems [4]. Clinical presentation varies from asymptomatic infection to a wide spectrum of features including fever, right upper quadrant abdominal pain, diarrhea, and weight loss [3, 4]. The most common extraintestinal manifestation is liver abscess and, rarely, pulmonary, cardiac, and brain involvement [1, 2].

Pulmonary amoebiasis is the second most common extraintestinal manifestation of *E. histolytica* infection, but often diagnosis is delayed, potentially leading to prolonged hospitalization and higher mortality [5, 6]. This case report describes lung empyema secondary to ruptured amoebic liver abscess complicated with venous thromboembolism and bronchopleural fistula. Pulmonary involvement accounts for only 2%–3% of extraintestinal amoebiasis but occurs more frequently in ALA, especially when ruptured [5]. Bronchopleural fistula can occur post‐thoracostomy or, rarely, as a manifestation of pulmonary amoebiasis [7, 8]. Venous thromboembolism is an uncommon yet potentially life‐threatening complication in ALA [9]. The existing literature on vascular complications in ALA provides minimal updated evidence, underscoring a significant knowledge gap regarding its current epidemiology and clinical implications. Awareness of these associations enables accurate diagnosis, early recognition, and treatment of ALA complications; therefore, improving patient outcomes.

## 2 Case Report

### 2.1 Clinical Presentation

A 26‐year‐old male with no previous illness presented with a three‐day history of sharp, pleuritic pain over the right hypochondrium. Further history revealed fever and dyspnoea for 2 weeks. There was no cough or diarrhea. He worked as a cleaner in a public lavatory. He had a hobby of river fishing and a history of kratom use.

On examination, the patient was alert but dehydrated. Vital signs on admission were as follows: heart rate, 139 beats/min; blood pressure, 120/80 mmHg; respiratory rate, 28 breaths/min; SpO_2_, 93% on room air; temperature, 37.6°C. He had reduced breath sounds in the right lower lung zone and tender hepatomegaly. There was no peritonism, splenomegaly, or lymphadenopathy.

### 2.2 Initial Investigations

**FIGURE 1:**
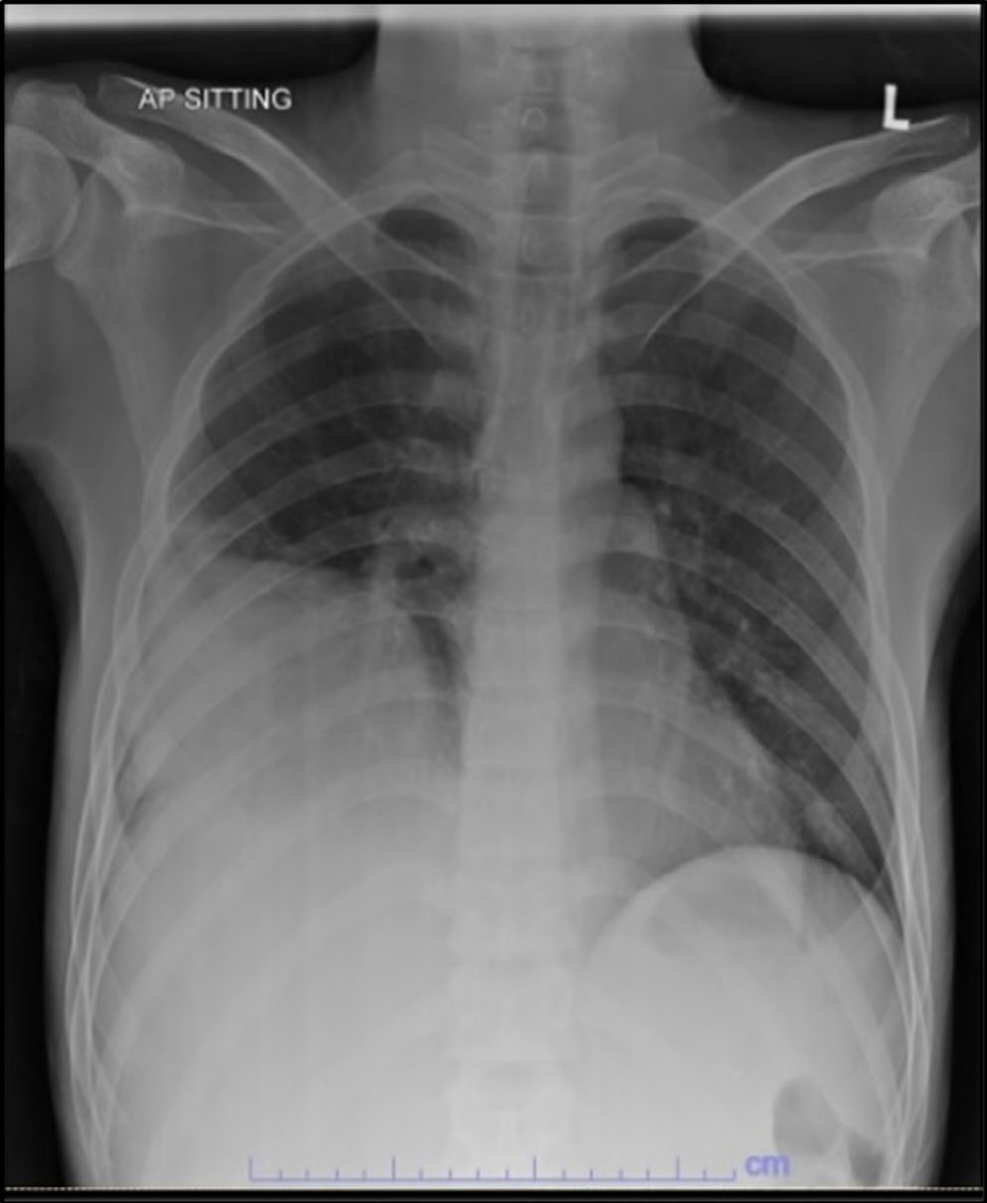
Chest radiograph. Lenticular heterogeneous opacity in the right middle and lower zone with obliterated costophrenic angle consistent with lung empyema.

**FIGURE 2:**
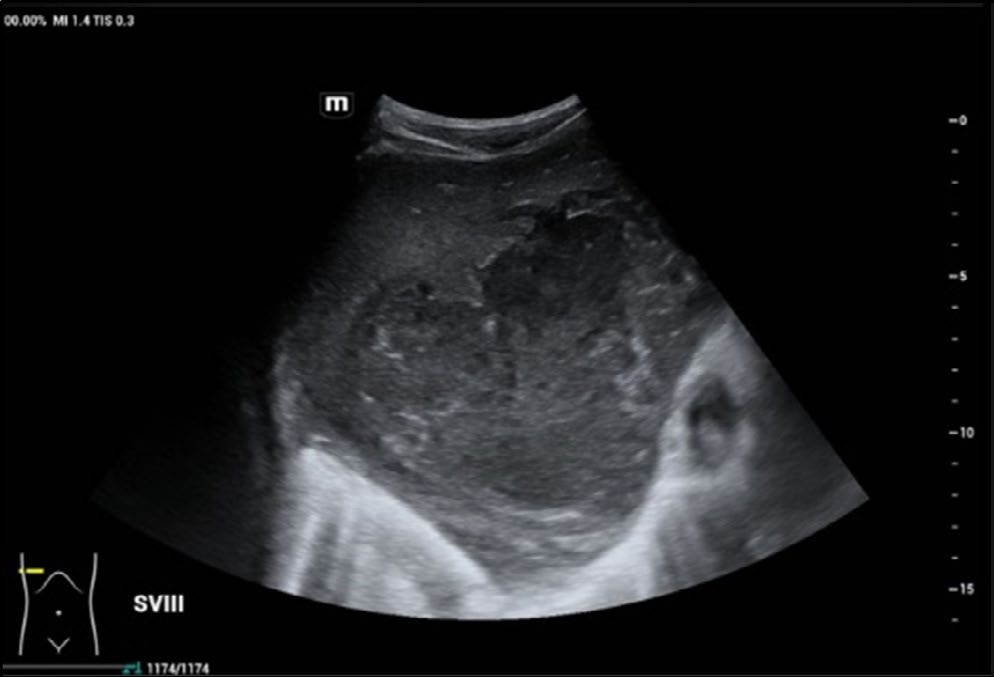
Hepatobiliary US. Hepatomegaly with a large lesion at segment V, VII, VIII measuring 11.5 × 14.2 × 15.0 cm. The heterogeneous and hypoechoic lesion is suggestive of partially liquefied liver abscess.

Full blood count showed normal white blood cell (WBC) count and microcytic hypochromic anemia with hemoglobin of 10.3 × 10^9^/L (Table 1). C‐reactive protein was raised at 267 mg/L. Liver profile showed elevated alkaline phosphatase (ALP), alanine transaminase (ALT), and aspartate transaminase (AST), bilirubin, and low albumin. International normalized ratio (INR) was prolonged. Prothrombin time (PT) and activated partial thromboplastin time (APTT) were normal. Renal profile showed hyponatremia with normal renal function. Human Immunodeficiency Virus antigen/antibody (HIV Ag/Ab), hepatitis B surface antigen (HBsAg), and anti‐hepatitis C virus (anti‐HCV) were negative. Fasting blood glucose was normal. Chest radiograph showed a lenticular heterogeneous opacity on the right lower zone (Figure 1). Hepatobiliary ultrasonography (US) showed a partially liquefied liver abscess (Figure 2).

**TABLE 1:** Summary of initial laboratory investigation.

Test	Unit	Values	Reference range
WBC	×10^9^/L	8.09	4–10
Hemoglobin	×10^9^/L	10.3	13–17
Platelet	×10^9^/L	250	150–410
C‐reactive protein	mg/L	267	< 10
Urea	mmol/L	6.7	2.8–8.1
Creatinine	μmol/L	71	44–80
Sodium	mmol/L	125	135–145
Potassium	mmol/L	4.9	3.5–5
Bilirubin	mmol/L	20	< 20
ALP	U/L	262	53–128
ALT	U/L	121	< 45
AST	U/L	165	< 35
Total protein	g/L	64	64–83
Albumin	g/L	24	35–52
Globulin	g/L	40	22–34
PT	s	36.7	9.1–12.5
INR		2.88	0.8–1.1
APTT	s	44.7	25.1–36.5
Fasting glucose	mmol/L	5.5	3.9–6

### 2.3 Clinical Course & Management

Intravenous ceftriaxone 2 g once daily and metronidazole 500 mg three times daily were commenced to treat the liver abscess with coverage of anaerobic microorganisms including amoebiasis. Ceftriaxone was then switched to ceftazidime 2 g three times daily to cover for *Burkholderia pseudomallei* , which is one of the common organisms causing liver abscesses and endemic in Malaysia. Following a two‐week course of ceftazidime with no evidence of melioidosis, the antibiotic was de‐escalated to oral amoxicillin/clavulanic acid 625 mg three times daily with a duration of 6 weeks to provide adequate coverage for pyogenic liver abscess.

**FIGURE 3:**
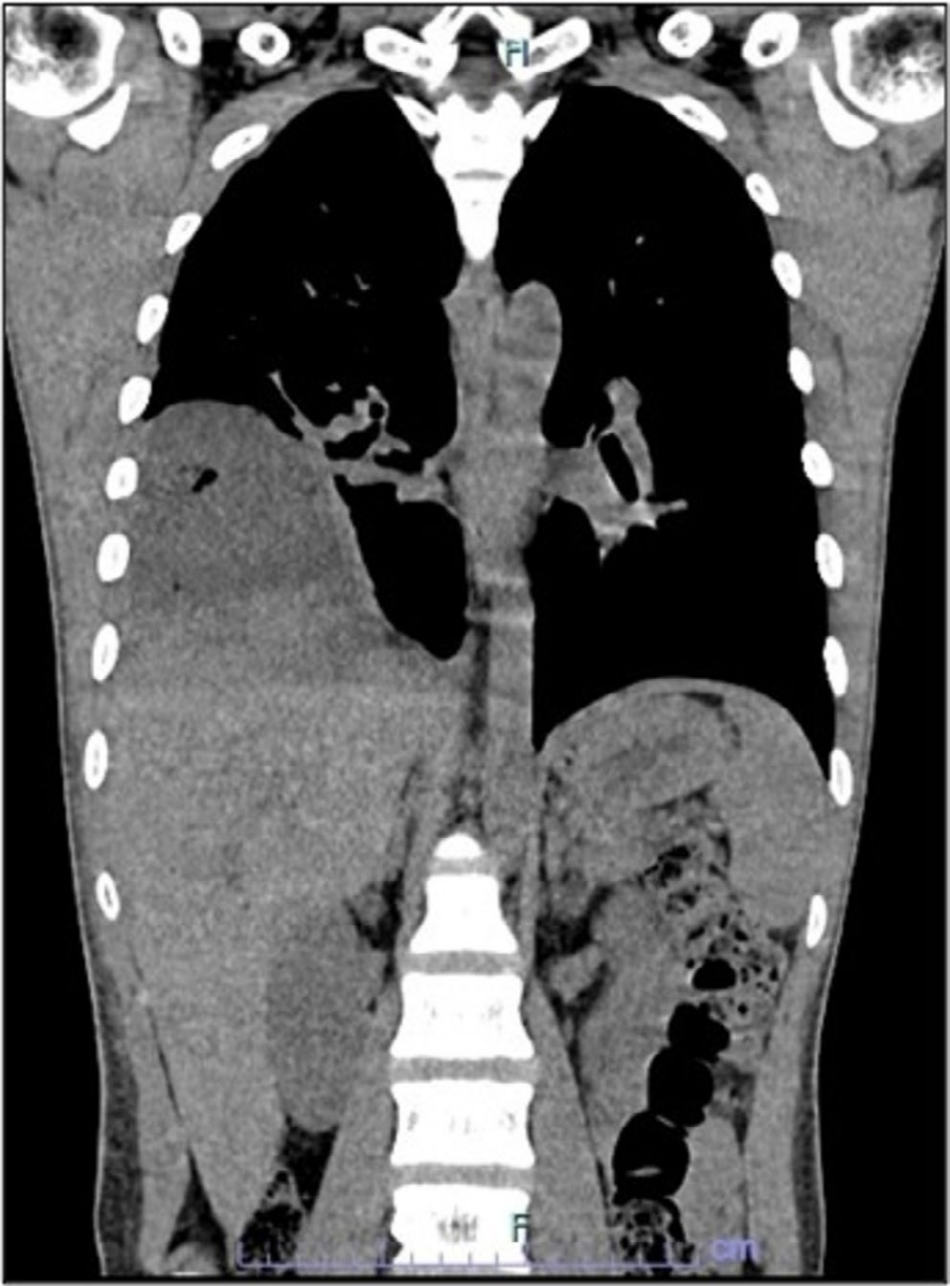
CT thorax and abdomen (coronal view). Large heterogeneous hypodense collection with ill‐defined margin in the liver extending to the periphery aspect of the right liver lobe and right intrathoracic region, measuring 11.9 × 9.8 × 10 cm, demonstrating ruptured right liver abscess with infra‐diaphragmatic intrathoracic extension.

Further imaging with computed tomography (CT) of the thorax and abdomen revealed a ruptured right liver abscess with infra‐diaphragmatic intrathoracic extension and right lung empyema (Figure 3). Inferior vena cava (IVC) and right hepatic vein thrombosis, and right pulmonary embolism were present, and a small ill‐defined lytic lesion was seen at T1 vertebra. The patient was commenced on warfarin for the treatment of venous thromboembolism.

Amoebiasis serology was later reported positive for IgG. Stool cultures and examination for ova and cysts were negative. The liver abscess was not amenable to drainage due to lack of a safe access window. The patient underwent insertion of a percutaneous pigtail thoracostomy catheter for drainage of right lung empyema, and the pus sample was positive for *E. histolytica* and giardiasis on polymerase chain reaction (PCR). A one‐week course of oral paromomycin 500 mg three times daily (25 mg/kg) was commenced for luminal clearance of amoebiasis.

Bacterial culture and sensitivity (C&S) on pus sample showed no growth. *Mycobacterium tuberculosis* (MTB) workup including acid‐fast bacilli (AFB) direct smear microscopy, culture, and PCR were negative (Table 2).

**TABLE 2:** Microbiology investigation.

Test	Result
Amoebiasis serology	IgG positive
Pus for MTB gene expert	Not detected
Pus for AFB direct smear	Negative
Pus for MTB C&S	No growth
Pus for MTB PCR	Not detected
Pus C&S	No growth
Amoebiasis PCR	*Entamoeba histolytica* and giardiasis positive
Blood C&S	No growth
16 S PCR	No bacteria detected
HIV Ag/Ab, HBsAg, anti‐HCV	Negative
Sputum C&S	No pathogen isolated
Melioidosis serology	Negative
Stool C&S	No growth
Stool examination for ova & cyst	Negative

### 2.4 Complications

Pigtail thoracostomy drainage showed continuous persistent air leakage after 1 week, suggestive of development of bronchopleural fistula. The final diagnosis was ruptured ALA with right pleural extension and empyema, complicated by venous thromboembolism and bronchopleural fistula.

### 2.5 Outcome

The patient underwent right video‐assisted thoracoscopy surgery (VATS) for visualization and repair of bronchopleural fistula following completion of antibiotics. Intraoperatively, the location of pig tail catheter tip was noted within the lung parenchyma, causing the air leak.

Repeat CT thorax and abdomen 2 months after completing treatment demonstrated a significant reduction in size of the right lateral intrathoracic and liver abscess. Due to the patient's non‐compliance to INR monitoring, warfarin was switched to rivaroxaban 20 mg OD for a duration of 6 months. Three monthly follow‐ups were arranged.

## 3 Discussion

This case demonstrates a presentation of empyema in amoebic liver abscess which was complicated by venous thromboembolism and bronchopleural fistula. Pulmonary complications occur in approximately 3% of patients with invasive amoebiasis, which is increased up to 40% in those with ALA [5]. 17.6% of pulmonary involvement by amoebiasis develops from empyema extending from the liver, which occurred in this patient due to extension of ruptured ALA through the diaphragm.

Amoebiasis is an important differential diagnosis in patients with liver abscess and should be investigated with appropriate tests. The laboratory diagnostic tests include parasitological (e.g., microscopy, culture), serological (e.g., enzyme‐linked immunosorbent assay (ELISA), indirect hemagglutination (IHA), latex agglutination), and molecular techniques [4]. The ‘gold standard’ is the microscopic observation of the parasite in stool, body fluid or tissue. However, stool examination is not generally sensitive compared to serological methods in extraintestinal amoebiasis. PCR‐based techniques show much higher sensitivity and specificity and are increasingly utilized for diagnosis [4, 5]. CT and magnetic resonance imaging (MRI) are widely performed to detect and accurately diagnose intestinal and extraintestinal amoebiasis [4, 6].

Management includes non‐pharmacological (e.g., basic hygiene practice and access to toilet and tap water) and pharmacological approaches [4, 6]. Pharmacological treatment is categorized into luminal and tissue amebicides [4, 5, 6]. Luminal amoebicides, e.g., paromomycin, diloxanide furoate, iodoquinol, act only on the intestinal lumen and are used to treat amoebic colitis. Tissue amebicides, e.g., nitroimidazoles (including metronidazole and tinidazole), are recommended in the treatment of invasive amoebiasis including ALA [1].

Metronidazole is widely used in the treatment of ALA, at 500 mg‐750 mg three times daily orally for 7 to 10 days [2, 3, 4, 5]. Alternatively, tinidazole 2 g OD PO can be prescribed for 3 days, with a 1% failure rate compared to 5% with metronidazole [3, 6]. The parasites can persist in the intestine in 40%–60% of patients receiving nitroimidazole. Therefore, initiation of a luminal agent, e.g., paromomycin (25–35 mg/kg/day in 3 divided doses for 7 days), following nitroimidazole is warranted to eliminate intraluminal cysts even if stool microscopy is negative [1, 2]. Oral iodoquinol 650 mg three times daily for 20 days is an alternative agent [3]. Diarrhea is a common side effect of paromomycin, and thus it should not be combined with nitroimidazoles, as this can make the assessment of response to therapy challenging [1, 2].

The incidence rate of rupture in ALA ranges from 6% to 40% [10]. For drainage of larger and incompletely liquefied ALA, percutaneous catheter drainage (PCD) is the preferred method over needle aspiration. In our patient, the liver abscess was not amenable to drainage due to a lack of a safe access window. The next management option was to place a percutaneous pigtail thoracostomy catheter to drain the lung empyema extending from the ruptured ALA through the diaphragm. Following this, the patient unexpectedly developed a bronchopleural fistula, which is a rare complication of thoracostomy [7]. Although Ochsner et al. reported a 3.1% incidence of bronchopleural fistula as a pulmonary manifestation of amoebiasis in 1936, recent literature provides little insight into its current incidence or clinical outcomes [8]. VATS approach has been used in the management of empyema secondary to ALA [11]. However, in our patient, this technique was chosen to repair the bronchopleural fistula due to the reduced risk of blood loss, postoperative pain, and length of hospital admission compared to open surgeries.

Our patient also had the presence of venous thromboembolism manifesting with IVC and right hepatic vein thrombosis and right pulmonary embolism, which are potentially life‐threatening sequelae of ALA that needed to be treated promptly. According to Arora et al., vascular complications in ALA include thrombosis in the IVC, hepatic vein, portal vein, right atrium, IVC obstruction, and pulmonary embolism [9]. Contemporary data on vascular complications in ALA are mainly based on case reports/series, with a significant gap in its current incidence, clinical profile, outcomes, and the risk versus benefit of prophylactic anticoagulant in the modern literature.

This case demonstrates the different characteristics of complicated ALA resulting in prolonged treatment and hospital stay. Interestingly, this patient had co‐infection with giardiasis although it is not a pathogenic organism for liver abscess. It is highly important for clinicians to identify the indications in ALA which require PCD or surgical management (Table 3) [12]. According to previous studies, the mortality rate in ruptured ALA has reduced from 22% in 1996 to 1% in 2021 with effective management strategies [12]. Presence of encephalopathy, hyperbilirubinaemia, hypoalbuminaemia, and increased abscess size are poor prognostic markers.

**TABLE 3:** Indications for medical therapy versus medical therapy + PCD/surgery in ALA cases [12].

Medical therapy	Medical therapy + PCD/surgery
Size < 10 cm * PCD is indicated in size 5–10 cm if no improvement after 3–5 days of medical therapy	Size > 10 cm Rim < 10 mm Left lobe Caudate lobe Type I ALA (acute aggressive) Unruptured ○ Vessel occlusion ○ Secondary infection Ruptured

## 4 Conclusion

This case highlights the pulmonary and vascular sequelae in complicated ALA, and underscores the importance of appropriate management, monitoring and timely treatment of complications. Physicians should maintain a high index of suspicion for invasive amoebiasis in patients presenting with liver abscess, particularly in endemic regions. Although pulmonary involvement is uncommon in invasive amoebiasis, it occurs more frequently in patients with ALA. Imaging, particularly CT, is an invaluable tool to detect these complications, as shown in this case and prior studies [5].

## Author Contributions

**Nur H. Ahnal:** data curation, writing – original draft, writing – review and editing. **Siti N. Mohd Nawi:** data curation, supervision, writing – review and editing. **Wan S. Wan Ghazali:** supervision, writing – original draft, writing – review and editing. **Alwi Muhd Besari:** conceptualization, supervision, writing – review and editing. **Hafsah Sazali:** data curation, investigation, writing – review and editing. **Fathin Hadi:** investigation, writing – review and editing.

## Funding

The authors have nothing to report.

## Ethics Statement

The authors have nothing to report.

## Consent

The authors have obtained a signed informed consent for publication from the patient.

## Conflicts of Interest

The authors declare no conflicts of interest.

## Data Availability

This manuscript does not report data generation or analysis. This manuscript is available as a preprint at Authorea, link to preprint: https://doi.org/10.22541/au.175690675.56669096/v1.

## References

[1] D.‐A. T. Shirley , L. Farr , K. Watanabe , and S. Moonah , “A Review of the Global Burden, New Diagnostics, and Current Therapeutics for Amebiasis,” Open Forum Infectious Diseases 5, no. 7 (2018): ofy161, 10.1093/ofid/ofy161. 30046644 PMC6055529

[2] R. Haque , C. D. Huston , M. Hughes , E. Houpt , and W. A. Petri, Jr. , “Amebiasis,” New England Journal of Medicine 348, no. 16 (2003): 1565–1573, 10.1056/NEJMra022710. 12700377

[3] S. L. Stanley , “Amoebiasis,” Lancet 361, no. 9362 (2003): 1025–1034, 10.1016/S0140-6736(03)12830-9. 12660071

[4] J. Nasrallah , M. Akhoundi , D. Haouchine , et al., “Updates on the Worldwide Burden of Amoebiasis: A Case Series and Literature Review,” Journal of Infection and Public Health 15, no. 10 (2022): 1134–1141, 10.1016/j.jiph.2022.08.013. 36155852

[5] S. M. Shamsuzzaman and Y. Hashiguchi , “Thoracic Amebiasis,” Clinics in Chest Medicine 23, no. 2 (2002): 479–492, 10.1016/s0272-5231(01)00008-9. 12092041

[6] J. Cooney , S. I. Siakavellas , P. L. Chiodini , et al., “Recent Advances in the Diagnosis and Management of Amoebiasis,” Frontline Gastroenterology 16 (2025): 37–50, 10.1136/flgastro-2023-102554. PMC12171400 40535508

[7] M. Kwiatt , A. Tarbox , M. J. Seamon , et al., “Thoracostomy Tubes: A Comprehensive Review of Complications and Related Topics,” International Journal of Critical Illness and Injury Science 4, no. 2 (2014): 143–155, 10.4103/2229-5151.134182. 25024942 PMC4093965

[8] A. Ochsner and M. DeBakey , “Pleuropulmonary Complications of Amebiasis: An Analysis of 153 Collected and 15 Personal Cases,” Journal of Thoracic and Cardiovascular Surgery 5 (1936): 225–258.

[9] B. Arora , L. Kakkar , and S. Mahal , “Vascular Complications of Amebic Liver Abscess—Computed Tomography Case Series With Review of the Literature,” Turkish Journal of Emergency Medicine 25, no. 2 (2025): 143–146, 10.4103/tjem.tjem_108_24. 40248476 PMC12002144

[10] R. N. Priyadarshi , R. Kumar , and U. Anand , “Amebic Liver Abscess: Clinico‐Radiological Findings and Interventional Management,” World Journal of Radiology 14, no. 8 (2022): 272–285, 10.4329/wjr.v14.i8.272. 36160830 PMC9453321

[11] J. A. Abello Vaamonde , E. G. White , A. M. López , and J. M. Lorenzo Silva , “Minimally Invasive Treatment of an Amebic Empyema Secondary to the Transdiaphragmatic Rupture of a Liver Abscess: A Case Report,” Journal of Surgical Case Reports 2022, no. 7 (2022): rjac334, 10.1093/jscr/rjac334. 35892123 PMC9307268

[12] R. Kumar , R. Patel , R. N. Priyadarshi , et al., “Amebic Liver Abscess: An Update,” World Journal of Hepatology 16, no. 3 (2024): 316–330, 10.4254/wjh.v16.i3.316. 38577528 PMC10989314

